# Postprandial metabolic effects of fructose and glucose in type 1 diabetes patients: a pilot randomized crossover clinical trial

**DOI:** 10.20945/2359-3997000000148

**Published:** 2019-07-11

**Authors:** Débora Lopes Souto, Érika dos Santos Lima, Joana Rodrigues Dantas, Lenita Zajdenverg, Melanie Rodacki, Eliane Lopes Rosado

**Affiliations:** 1 Universidade Federal do Rio de Janeiro Universidade Federal do Rio de Janeiro Instituto de Nutrição Josué de Castro Departamento de Nutrição e Dietética Rio de Janeiro RJ Brasil Universidade Federal do Rio de Janeiro (UFRJ), Instituto de Nutrição Josué de Castro, Departamento de Nutrição e Dietética, Rio de Janeiro, RJ, Brasil; 2 Universidade Federal do Rio de Janeiro Universidade Federal do Rio de Janeiro Departamento de Medicina Interna Rio de Janeiro RJ Brasil Universidade Federal do Rio de Janeiro (UFRJ), Departamento de Medicina Interna, Seção de Diabetes e Nutrologia, Rio de Janeiro, RJ, Brasil

**Keywords:** Type 1 diabetes, fructose, glucose, malondialdehyde, uricaemia

## Abstract

**Objective:**

To test the influence of oral fructose and glucose dose-response solutions in blood glucose (BG), glucagon, triglycerides, uricaemia, and malondialdehyde in postprandial states in type 1 diabetes mellitus (T1DM) patients.

**Subjects and methods:**

The study had a simple-blind, randomized, two-way crossover design in which T1DM patients were selected to receive fructose and glucose solutions (75g of sugars dissolved in 200 mL of mineral-water) in two separate study days, with 2-7 weeks washout period. In each day, blood samples were drawn after 8h fasting and at 180 min postprandial to obtain glucose, glucagon, triglycerides, uric acid, lactate, and malondialdehyde levels.

**Results:**

Sixteen T1DM patients (seven men) were evaluated, with a mean age of 25.19 ± 8.8 years, a mean duration of disease of 14.88 ± 4.73 years, and glycated hemoglobin of 8.13 ± 1.84%. Fructose resulted in lower postprandial BG levels than glucose (4.4 ± 5.5 mmol/L; and 12.9 ± 4.1 mmol/L, respectively; p < 0.01). Uric acid levels increased after fructose (26.1 ± 49.9 µmol/L; p < 0.01) and reduced after glucose (-13.6 ± 9.5 µmol/L; p < 0.01). The malondialdehyde increased after fructose (1.4 ± 1.6 µmol/L; p < 0.01) and did not change after glucose solution (-0.2 ± 1.6 µmol/L; p = 0.40). Other variables did not change.

**Conclusions:**

Fructose and glucose had similar sweetness, flavor and aftertaste characteristics and did not change triglycerides, lactate or glucagon levels. Although fructose resulted in lower postprandial BG than glucose, it increased uric acid and malondialdehyde levels in T1DM patients. Therefore it should be used with caution. ClinicalTrials.gov registration: NCT01713023.

## INTRODUCTION

Sweetness is considered one of the most powerful determinants of food consumption (1). Among sugars, fructose has raised interest because it results in lower postprandial glucose concentrations than the ingestion of isocaloric amounts of other carbohydrates. Therefore, fructose used as a sweetening agent in the diet of patients with diabetes may have a definite advantage (2). Nevertheless, fructose has potentially harmful effects on other aspects of metabolism such as weight gain, cardiovascular diseases, and gout (3,4). Thus, the effects of fructose intake on diabetes complications must be studied.

Previous studies that included type 1 diabetes mellitus (TIDM) patients have examined the effect of this sugar given as part of a meal or as part of the sucrose molecule (2). The combination of the fructose and glucose produced a synergistic increase in glycogen accumulation in hepatocytes, while, when the fructose given alone contribute to increase triglycerides, lactate, pyruvate, and oxidative stress levels (3,4).

In the absence of an international consensus on what is adequate or excessive fructose intake, we conducted a simple-blind, randomized, two-way crossover pilot study to examine the postprandial influences of an oral fructose and glucose tolerance test in the blood glucose, triglycerides, uricaemia, and malondialdehyde levels in T1DM patients.

## SUBJECTS AND METHODS

### Subjects

T1DM patients were recruited through poster advertisements or invited during routine medical appointment at the Clementino Fraga Filho University Hospital, Brazil (between October 2013 and December 2015) by an author.

All volunteers were diagnosed with T1DM according to the American Diabetes Association criteria (5), and used the subcutaneous insulin infusion system or multiple insulin injections with long-acting insulin analogues (glargine, detemir or degludec) combined with a short-acting analogs (aspart, lispro or glulisin).

Exclusion criteria were hypertension, renal or hepatic impairment, rheumatologic disease, evidence of diabetic complications, delayed gastric emptying or gastroparesis symptoms, use of anti-lipidemic, antibiotics or anti-inflammatory drugs or antidiabetic medications, smoking, alcoholism, visual difficulty, and parental history of other types of diabetes. Patients with disease duration less than three years were also excluded.

The sample size and selection by convenience. All participants signed an informed consent, and the study was approved by the Ethical Committee (Institutional Review Board, protocol 151/11) and was registered at ClinicalTrial.gov (NCT 01713023).

### Study design

This was a pilot single-blind, randomized, two-way crossover study in which sixteen TIDM patients were randomly selected to receive either glucose or fructose in two separate oral dose-response solution, with 2-7 weeks washout period.

One day before each study day, the researcher contacted the patient to ask about events that could influence the results (e.g. infection, flu, fever, hypoglycemia) or if they reported significant deviations from their usual life patterns. The test was rescheduled in those situations. They were also instructed to refrain from alcohol consumption and any unusual exercise and activity 24h before.

All participants maintained their usual long-acting insulin analogues dosage, while theirs dose of the short-acting analogs were suspended in the morning on each study day.

On each study day, the patient arrived at the Clementino Fraga Filho University Hospital at 7-8h A.M. after 8h overnight fast. Upon arrival, anthropometric variables, capillary blood glucose (CBG) and venous blood glucose samples were collected.

A number generator was used to randomly select the order of each intervention (oral fructose or glucose tolerance test solution) to be assigned to each the patient.

All patients were instructed to drink each solution within three min and then complete questionnaires assessing the sweetness and palatability of the solution (6). CBG were collected at 30, 90, 120, and 180 min after consumption of the solutions. The second venous blood sample was drawn 180min after the volunteer received the test solution. As another authors (7-9), we have chosen these timepoints (T_0_-T_180_) to evaluate the glycemic response to carbohydrates because the total carbohydrates digestion and absorption occurs within three hours (180 min).

At the end of each intervention, the volunteers received a dose of the short-acting analogs, considering the postprandial capillary blood glucose, insulin sensitivity and the amount of carbohydrate in the snack that was offered.

### Oral fructose and glucose tolerance test solutions and assessment of sweetness and palatability

Oral glucose and fructose dose-response solutions were composed by 75g of white crystalline powder of fructose (Lowçucar, LightSweet, Brazil) or white crystalline powder dextrose (Glutol, Laborclin, Brazil) dissolved in 200 mL of mineral-water at ambient temperatures (21-25ºC or 73-77ºF).

The questionnaire described by Crapo and cols. (6) was used to quantify the sweetness and palatability of solutions.

### Dietary intake and physical activity assessments

Before starting the intervention, patients filled in a 3-day (2 weekdays and 1 weekend day) food record. During those three days, each food and drinks consumed had to be documented to allow quantitative estimation of dietary intake. Data were then entered into the DietPró 5.5i nutrition software (version 2010, Brazil) to convert the amount of food eaten into individual nutrients and the mean daily energy and nutrient intake for each patient was calculated. The 3-day energy and nutrient intakes were averaged to obtain a mean daily energy and nutrient intake for each patient.

The short-form of the international physical activity questionnaire was used to access the regular physical activity (10).

### Capillary blood glucose assessments

In each study day, CBG were measured by fingerpick with the use of a glucometer (Accu-Chek Active; Roche Diagnostics, Brazil). The same glucometer was used for the same patients for all two examination-day. The CBG samples were obtained at baseline and 30, 90, 120, 180 min after consumption of each oral sugars dose-response solutions.

### Anthropometric measurements

Body mass index (BMI) was calculated as body weight in kilograms divided by the square of height in meters (11). Waist circumference was determined as the average of two measurements calculated to the nearest 0.1 cm midway between the lower rib margin and the iliac crest after a normal expiration (12).

Body composition was measured hand-to-foot by tetrapolar bioelectrical impedance (Bioimpedance Analyzer 450, Biodynamics Corporation. Shoreline, WA, USA) (13).

### Laboratory analysis

Screening blood tests performed at baseline included glycated hemoglobin (HbA1c), glucagon, fructosamine, creatinine, aspartate aminotransferase (AST), alanine aminotransferase (ALT), and cholesterol profile.

Glucose, triglycerides, glucagon, uric acid, lactate, and malondialdehyde levels were measured at baseline and 180 min after the intake of the OGTT and OFTT.

HbA1c was measured by the high-performance liquid chromatography (Variant II; Bio-Rad Laboratories^®^, USA). Fructosamine was measured by enzymatic colorimetric method (Roche Diagnostics^®^, UK).

Glucose, total cholesterol, HDL, and triglycerides were measured by an enzymatic colorimetric method (Labtest Diagnostic^®^, Brazil). LDL was calculated (14).

AST and ALT were determined using the ultraviolet kinetic method (Labtest Diagnostic^®^, Brazil).

Serum creatinine was measured by a direct colorimetric method (Labtest Diagnostic^®^, Brazil) to calculate creatinine clearance by using the Cockcroft–Gault formula (15).

Uric acid and lactate were measured by kinetic enzymatic method, with kits from Labtest Diagnostic^®^ (Brazil) and Roche Diagnostics^®^ (UK), respectively.

Glucagon and malondialdehyde levels were determined using the enzyme-linked immunosorbent assay (Life Science Inc^®^, USA). Malondialdehyde limit of detection ranged between 40-130 ng/L.

### Statistical analysis

Statistical analyses were performed in SPSS software (version 17.0; SPSS Inc, Chicago, IL, USA). A p-value < 0.05 was considered statistically significant.

Qualitative variables were described as frequency, whereas quantitative variables were described as the mean ± standard deviations (SD) and 95% CI.

The Mann-Whitney test was used for between-group comparison and the Wilcoxon test was used to compare the effects of tests in each group. Spearman correlation and linear regression were used to evaluate laboratory analysis interaction with anthropometric variables.

Time course of CBG were analyzed with repeated measures analysis of variance two-way ANOVA.

## RESULTS

### Characteristics of the study group at baseline

Sixteen TIDM patients (7 men) were evaluated, and their characteristics are presented in Table 1. All were in a basal-bolus plan, with 12 using multiple daily injections and 4 with a subcutaneous infusion system (details in the supplementary Table 1).

**Table 1:** Baseline characteristics of the study group

Sex (female/male)	7/9
Age (years)	25.19 ± 8.8 (18 – 54)
Duration of type 1 diabetes (years)	14.88 ± 4.73 (7 – 27)
Body mass index (kg/m^2^)	24.7 ± 3.66 (17.6 – 30.3)
Waist circumference (cm)	84.45 ± 8.24 (67.50 – 96.50)
Body fat (%)	24.05 ± 6.03 (14.30 – 32.90)
Lean body mass (%)	73.05 ± 11.19 (49.10 – 85.70)
Total body water (L)	39.06 ± 7.88 (28.90 – 51.00)
Glycosylated hemoglobin (%)	8.13 ± 1.84 (5.50 – 12.00)
Fructosamine (mcmol/L)	391.94 ± 87.36 (275.00 – 539.00)
Creatinine (µmol/L)	67.18 ± 24.75 (26.52 – 114.92)
Creatinine clearance (mL/s)	3.37 ± 1.29 (1.72 – 6.37)
Total cholesterol (mmol/L)	4.26 ± 1.20 (2.79 – 6.41)
HDL (mmol/L)	1.39 ± 0.46 (0.80 – 2.37)
LDL (mmol/L)	2.48 ± 0.78 (1.26 – 3.92)
Aspartate aminotransferase (units/L)	22.78 ± 8.65 (9.00 – 40.00)
Alanine aminotransferase (units/L)	16.47 ± 5.58 (10.00 – 31.00)
Basal insulin dose (units/kg/d)	0.52 ± 0.22 (0.25 – 1.13)
Total insulin dose (units/kg/d)	0.95 ± 0.33 (0.47 – 1.75)

Data are means ± standard deviations (95% CI).

The baseline characteristics of the study group were measured at the first study day. However, when the washout period exceeds fifteen days (7 patients), the baseline characteristics were revalued on the second study day. Baseline characteristics did not change during the washout period (details in the supplementary Table 2).

**Table 2:** Sweetness and palatability characteristics of oral fructose and glucose dose-response solution

	Fructose	Glucose	p-value*
Sweetness	0.85 ± 0.92 (0.00 – 3.00)	1.03 ± 1.10 (0.00 – 3.00)	0.78
Flavor	6.23 ± 2.30 (2.70 – 10.00)	5.48 ± 2.89 (0.80 – 9.70)	0.41
Dilution	8.93 ± 1.79 (3.20 – 10.00)	5.20 ± 2.49 (2.10 – 9.80)	< 0.01
Aftertaste	3.18 ± 2.96 (0.00 – 9.90)	3.45 ± 2.64 (0.00 – 7.90)	0.61

Data are means ± standard deviations (95% CI).

* p-values were derived by Mann-Whitney test.

Although the mean HbA1c values was slightly above target (< 7%), the other routine tests were mostly within the treatment goals set by the American Diabetes Association (16) (Table 1).

Patients presented an usual normoproteic, normoglycidic, hyperlipidic diet and an adequate fiber intake (17) (details in the supplementary Table 3). In addition, most patients were classified as active (details in the supplementary Table 4).

**Table 3:** Capillary blood glucose concentrations (mmol/L) at baseline and over time the oral sugars dose-response solution

	Baseline	Time after intake (minutes)				p-value^†^

		30	90	120	180	
Fructose	8.16 ± 3.00 (2.55 – 11.98)	10.01 ± 3.47 (3.99 – 15.53)	12.68 ± 4.30 (3.32 – 19.20)	13.44 ± 4.61 (3.49 – 19.36)	12.37 ± 5.30 (3.32 – 25.19)	< 0.01
Glucose	6.88 ± 3.07 (3.38 – 12.54)	12.49 ± 6.00 (6.10 – 31.19)	21.11 ± 3.41 (15.59 – 27.41)	17.45 ± 4.00 (14.85 – 22.75)	19.22 ± 4.60 (8.82 – 27.80)	< 0.01
p-value*	0.23	0.37	< 0.01	< 0.01	< 0.01	< 0.01

* p-values were derived by Mann-Whitney test.

^†^ p-values were analyzed with repeated measures analysis of variance two-way ANOVA.

**Table 4:** Effects of oral sugar dose-response solutions on baseline (T0) and postprandial (T180) laboratory tests

	Fructose			Glucose			p-value^‡^	p-value^§^
	
	Levels*	Δ*	p-value^†^	Levels*	Δ*	p-value^†^		
Glucose (mmol/L) T_0_	7.97 ± 3.29 (1.94 – 13.04)	4.48 ± 5.57 (-2.71 – 20.70)	< 0.01	6.70 ± 3.05 (3.38 – 11.93)	12.98 ± 4.16 (4.38 – 19.75)	< 0.01	0.25	< 0.01
Glucose (mmol/L) T_180_	12.47 ± 5.69 (3.05 – 24.64)			19.65 ± 5.22 (6.32 – 28.91)			< 0.01	
Triglycerides (mmol/L) T_0_	3.56 ± 1.62 (1.60 – 7.54)	0.20 ± 0.66 (-0.61 – 1.94)	0.32	3.85 ± 1.70 (1.60 – 6.77)	-0.24 ± 1.45 (-3.44 – 3.21)	0.20	0.54	0.07
Triglycerides (mmol/L) T_180_	3.77 ± 1.94 (1.83 – 9.49)			3.60 ± 2.17 (0.77 – 8.10)			0.65	
Lactate (mmol/L) T_0_	1.17 ± 0.54 (0.34 – 2.24)	0.13 ± 0.58 (-1.06 – 1.03)	0.21	1.14 ± 0.34 (0.62 – 1.77)	-0.14 ± 0.39 (-0.72 – 0.92)	0.07	0.95	0.05
Lactate (mmol/L) T_180_	1.30 ± 0.69 (0.36 – 2.96)			0.99 ± 0.35 (0.57 – 1.87)			0.16	
Glucagon (ng/L) T_0_	126.93 ± 64.21 (23.45 – 269.54)	13.04 ± 43.76 (-83.00 – 119.64)	0.10	131.46 ± 35.73 (65.10 – 194.05)	-11.06 ± 34.04 (-72.69 – 40.36)	0.43	0.52	0.10
Glucagon (ng/L) T_180_	139.98 ± 53.12 (50.43 – 229.77)			120.40 ± 34.22 (49.13 – 171.42)			0.20	
Uric acid (µmol/L) T_0_	3.48 ± 2.17 (1.70 – 10.90)	0.44 ± 0.84 (-0.60 – 3.20)	< 0.01	2.90 ± 0.85 (1,50 – 4,30)	-0.23 ± 0.16 (-0.50 – 0.00)	< 0.01	0.66	< 0.01
Uric Acid (µmol/L) T_180_	3.93 ± 2.93 (1.70 – 14.10)			2.66 ± 0.82 (1.50 – 3.80)			0.07	
Malondialdehyde (µmol/L) T_0_	10.06 ± 2.06 (6.55 – 12.86)	1.40 ± 1.60 (-0.76 – 4.73)	< 0.01	11.78 ± 1.71 (8.22 – 14.02)	-0.27 ± 1.60 (-2.70 – 2.60)	0.40	0.03	< 0.01
Malondialdehyde (µmol/L) T_180_	11.46 ± 1.72 (8.55 – 14.79)			11.50 ± 1.30 (9.58 – 13.91)			0.82	

^*^ Means ± standard deviations (95% CI).

Delta (Δ): measured as the difference between postprandial and baseline values after each solution.

^†^ p-values were derived by analysis of covariance with basal and postprandial values after each solution (Wilcoxon signed rank test).

^‡^ p-values were derived by Mann-Whitney test to compare basal and postprandial values between the oral fructose and glucose dose-response solutions.

^§^ p-values were derived by Mann-Whitney test to compare the difference of delta between postprandial (T_180_) and baseline (T_0_) values between the oral fructose and glucose dose-response solutions.

Regular physical activity and usual dietary intake did not change during the washout period.

### Sweetness and palatability characteristics of solutions

Both solutions were well tolerated by all 16 TIDM patients, and no adverse events (like sickness, diarrhea or nausea) were observed.

Solutions had similar sweetness, flavor and aftertaste characteristics. However, oral glucose dose-response solution presented a lower dilution rate, while oral fructose dose-response solutions were readily diluted to homogeneous solutions (p <0.01) (Table 2).

### Effects of oral fructose and glucose tolerance test solutions in the capillary blood glucose

Table 3 shows the CBG concentrations over time after drinking the oral sugars dose-response solutions.

Baseline CBG before intake the solutions were almost identical (p = 0.23).

The maximum difference in CBG concentrations between the baseline and after the administration of the solutions were observed at 120 min and 180 min, for oral fructose and glucose dose-response solutions, respectively. Two-factor repeated-measures ANOVA revealed a difference over time after the solutions (F = 567.90; p < 0.01).

### Postprandial changes in laboratory tests

The influences of the test solutions in the laboratory tests are presented in Table 4.

At baseline, a few patients presented hypoglycemic episodes (glucose < 3.8 mmol/L) on fructose (n = 3; 18.7%) and oral glucose dose-response solution (n = 1; 6.2%). Hyperglycemic episodes (≥ 9.99 mmol/L) also occurred before the fructose (n = 5; 31.2%) and oral glucose dose-response solution (n = 1; 6.25%).

Both solutions increased the postprandial plasma glucose levels (p < 0.01). However, glucose resulted in greater postprandial glycaemia when compared to fructose (p < 0.01).

Serum uric acid levels increased after fructose (p < 0.01) but reduced after oral glucose dose-response solution (p < 0.01). The difference between postprandial and baseline values (delta) of uric acid also showed differences between solutions (p < 0.01).

Glucagon, triglycerides and lactate levels did not change after oral sugar dose-response solutions (fructose: p = 0.10, p = 0.32, and p = 0.21; glucose: p = 0.43, p = 0.20, and 0.07 respectively).

At baseline, malondialdehyde serum levels were higher in patients before the oral glucose dose-response solution than in those that received oral fructose dose-response solution (p < 0.01). However, we observed that the variation between postprandial and baseline values showed that glucose intake did not result in malondialdehyde elevation (p = 0.40) while fructose solution increased the malondialdehyde levels (p < 0.01). In addition, comparing the variations in malondialdehyde before and after the intake of the solutions also differed between fructose (p < 0.01), confirming the increase in malondialdehyde levels after oral fructose solution.

The relative contributions of baseline characteristic differences between patients were assessed by linear regression and Spearman correlation. The duration of T1DM and HbA1c levels were not associated any of the laboratory variables (p > 0.05). BMI (p = 0.38; r = 0.38; p = 0.03), body fat (r = 0.37; p = 0.38; p = 0.03), and waist circumference (r = 0.43; p = 0.38, p = 0.02) as were independently associated with lactate levels.

## DISCUSSION

In this study we showed that fructose solution resulted in lowe r blood glucose levels than oral glucose dose-response solution in T1DM patients but increased uric acid and malondialdehyde levels. This was the first clinical trial that assessed the influences of monosaccharides in malondialdehyde levels in T1DM patients, which is a potential biomarker for oxidative stress. Oxidative stress is implicated in the pathogenesis of chronic diabetic complications (18).

The smaller rise in plasma glucose levels with the fructose solution than with the glucose solution shown in this analysis was expected and consistent with other studies (2,19). There are only a few clinical trials that assessed the effect of glucose and fructose intake in metabolic control of T1DM patients. These studies quantified the plasma glucose responses for several different food into a “glycemic index”. The “glycemic index” was defined as the increase in plasma glucose area from zero to 120min after ingestion of 50g of available carbohydrate from a test food compared with 50g of carbohydrate from a reference food (white bread or glucose). For fructose, a particularly “low glycemic index” was described, with a smaller postprandial increase in plasma glucose than with other carbohydrates (7).

This response is similar in T1DM and type 2 diabetes patients (20). Even though fructose and glucose have similar caloric content, they have different response on the carbohydrate metabolism. This should be partially explained by fructose clearance by the liver in an insulin independent manner as well as the activation of different pathways than other carbohydrates (3).

There has been some concern over the possibility of fructose induced hypertriglyceridemia and this question is particularly relevant for patients with diabetes, in which hypertriglyceridemia is the most common lipid abnormality (2,3). Fructose is metabolized in the liver by phosphorylation on the 1-position, a process that bypasses the rate-limiting phosphofructokinase step. Thus, hepatic metabolism of fructose favors lipogenesis, and may change circulating lipids (3).

High levels of fructose are converted to acetyl-CoA in the liver in a non-regulated manner, increasing the de novo lipogenesis, which facilitates the triglycerides production. Most of the studies evaluated its long-term use (from 2-4 weeks) of fructose in type 2 diabetes patients with overweight or obesity. These studies found a significant increase of the triglycerides levels, some with different responses according to gender, age and amount of fructose ingested (21,22). On the other hand, in our study, the acute effect of one single large load of fructose did not raise triglycerides levels. Fructose might have had a lower effect on the lipid metabolism in our patients because they were younger, and the majority was eutrophic, without features of insulin resistance. In addition, the fact that it was an acute exposure to fructose instead of chronic intake could also have influenced the results.

In this study, the effects of sugar solutions in plasma glucagon levels were different from previous analyses. In contrast to observed by Kramer and cols., we verified no changes in glucagon levels after OGTT in T1DM patients. However, the differences in study design may explain the contrary results. Our patients were treated with subcutaneous long-acting insulin analogs or subcutaneous infusion system, while in the study performed by Kramer and cols. the insulin was administered intravenously (23).

No other studies evaluated glucagon levels in T1DM patients after a fructose overload. Although a previous study showed that the glucagon secretion was unresponsive to intravenous infusion of fructose during hypoglycemia in healthy subjects, we found a postprandial increase in glucagon levels after the intake of the OFTT in T1DM patients (24). This difference is probably due to an abnormal regulation of glucagon secretion in T1DM patients (25), and different routes of administration of fructose (23).

In contrast to healthy individuals, who have a reduction in glucagon levels after meals, previous studies have found that not only the α-cell secretory reserve is preserved by the ongoing autoimmune process in T1DM, but also that these patients have an inappropriately high glucagon response to meals (26,27).

Moreover, glucagon is implicated in the pathogenesis of diabetic ketoacidosis (28,29). As fructose acutely increased glucagon levels in T1DM patients, it is possible that the chronic use of this sugar as a sweetener might increase the risk of diabetes ketoacidosis in these individuals.

The current study found a reduction in uric acid levels after OGTT and an elevation after the OFTT. No other studies assessed the effect of oral sugar dose-response solutions in uricaemia of T1DM patients. However, previous studies in healthy individuals (30) and in type 2 diabetes patients (31) have shown similar results. We hypothesize that hyperglycemia with glycosuria resulted in an increase in the renal excretion of urates, leading to lower uric acid levels (32). Although we did not find any significant correlation between plasma glucose levels and uric acid, it is a consensus that glycosuria occurs when the blood glucose concentration is greater than 9.99 mmol/L (33).

In contrast to glucose, we verified an increase of the uric acid and malondialdehyde levels after OFTT. This probably occurs because the one key difference between fructose and glucose is in the initial carbohydrate metabolism. Fructose is metabolized in the liver by fructokinase, which uses ATP to phosphorylate fructose to fructose-1-phosphate. Unlike hexokinases, which phosphorylate glucose and have a negative feedback system to prevent excessive phosphorylation, fructokinase phosphorylates fructose as rapidly as it can, and this commonly leads to intracellular phosphate depletion. Lower intracellular phosphate levels result in the activation of AMP deaminase, which converts the AMP to inosine monophosphate, and subsequently in hypoxanthine. The enzyme xanthine oxidase catalyzes the oxidation of hypoxanthine to xanthine and then to uric acid, which plays a crucial role in oxidative stress because the action of xanthine oxidase produces superoxide radicals that are derived from reactive oxygen species (34). However, uric acid may contribute to more than 50% of the antioxidant capacity of the blood, and it also has a direct effect on the inhibition of free radicals such as peroxynitrite radical and peroxyl (35).

The effect of fructose in the activity of xanthine oxidase might have increased the oxidative stress and induced the generation of malondialdehyde. Serum malondialdehyde and urinary F2-isoprostane are the most frequently measured biomarkers of oxidative stress, and their levels are increased in T1DM patients. Malondialdehyde is generated by both lipid oxidation and as a by-product of prostaglandin and thromboxane synthesis. Advanced glycation products generated in the hyperglycemic state stimulate lipolysis, which increases the production of malondialdehyde (36).

T1DM patients seem to have an increased susceptibility to oxidative stress due to decreased antioxidant defense caused by hyperglycemia and glucose variability (18). Fructose intake induces the activity of xanthine oxidase, which may have clinical relevance because this enzyme has been involved in the pathogenesis of oxidative stress and several diabetic chronic complications (37). Studies have described that high malondialdehyde levels is associated with glomerular hyperfiltration (38) and sympatic disfunction (39) in T1DM patients. Therefore, the use of fructose could be potentially harmful, leading to an increase of oxidative stress and turning patients more prone to the development of chronic complications. Further studies with the chronic use of fructose as a sweetener in T1DM patients are necessary to address this hypothesis.

Our patients reported that fructose was readily diluted to homogeneous solutions. In addition, both sugar solutions had similar sweetness, flavor and aftertaste characteristics. Other authors suggested that the fructose is more soluble in water, and sweeter than glucose (2-4). However, the perceived intensity for sugar taste perception differs among individuals, based on their prior experiences, age, gender, genetic taste, and diseases. Another study (40) has also showed that the taste sensation was reduced in T1DM patients, and the decreased taste acuity may be an important factor in the perception of sweet taste.

Our study has limitations. First, because the stringent eligibility criteria required for participation in our crossover study, the sample was selected by convenience. Thus, the results could not represent what would happen in the entire population of T1DM (41). Secondly, we did not evaluate the post challenge glycosuria in the study group. This test would be helpful to identify if the decreased serum uric acid levels are a result of the increased glucose excretion (34). As a third limitation, the duration of T1DM, HbA1c levels, BMI, body fat, and waist circumference differed widely between patients included in the study, but this was a crossover study. Therefore, these differences did not interfere in the comparison between the two solutions. The fourth limitation included the hypo- and hyperglycemic episodes at baseline. Although the fasting glucose was not adequate at baseline, the crossover study design reduced the influence of confounding covariates because each crossover patient serves as his or her own control (42). The last limitation was that we included only an acute evaluation of the effect of glucose and fructose in metabolic variables and a long-term follow-up would also be important to determine the benefits and the risks of fructose in T1DM patients.

In conclusion, fructose intake elicited a lower blood glucose response than glucose and that did not induce alterations of the triglycerides, lactate or glucagon levels.

However, the intake a large amount of fructose resulted in an increase of uric acid and malondialdehyde levels. Therefore, T1DM patients that use fructose as a regular sweetener should be aware that this practice might impact their susceptibility to hyperuricemia and stress oxidative, which might have clinical implications.

Further clinical studies will be needed to better understand the mechanisms, specifically regarding the uric acid and malondialdehyde metabolism and their potential role in diabetic microvascular complications. Therefore, future studies will be able to evaluate the fructose consumption, and to compare fructose and glucose with other sweetening agents (for example sucrose, honey, agave) in these patients.

Author contributions: DLS and ESL conceived, performed and coordinated the study. LZ, MR, and JRD helped in collecting data and contributed to draft the manuscript. ELR participated in design and coordination. All authors read and approved the final manuscript.
